# Collodion

**Published:** 1848-05

**Authors:** 


*Collodion.*—This is the name applied to the new preparation proposed as a substitute for adhesive plaster. The term is derived from *kolla*, glue; and *eidos*, resembling. The two claimants for the discovery of its application to surgical objects, are contending for the honor in the columns of the Boston Medical Journal. We suppose there is no difficulty in the manufacture of the article, but we perceive that both combatants have forgotten, in the heat of controversy, to state the process of which it is prepared. We are not so uncharitable as to suspect there is any disposition to keep the process secret.

